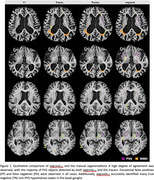# segcsvd_PVS_: A convolutional neural network‐based tool for quantification of enlarged perivascular spaces (PVS) on T1‐weighted images

**DOI:** 10.1002/alz70856_104470

**Published:** 2025-12-26

**Authors:** Erin Gibson, Joel Ramirez, Lauren Abby Woods, Stephanie Berberian, Julie Ottoy, Christopher JM Scott, Vanessa Yhap, Fuqiang Gao, Roberto Duarte, Maria del C. Valdes Hernandez, Anthony E. Lang, Carmela Tartaglia, Sanjeev Kumar, Malcolm Binns, Robert Bartha, Sean Symons, Richard H. Swartz, Mario Masellis, Navneet Singh, Bradley J. MacIntosh, Joanna M Wardlaw, Sandra E. Black, Andrew Lim, Maged Goubran

**Affiliations:** ^1^ Dr. Sandra E. Black Centre for Brain Resilience and Recovery, LC Campbell Cognitive Neurology, Hurvitz Brain Sciences Program, Sunnybrook Research Institute, University of Toronto, Toronto, ON, Canada; ^2^ Dr. Sandra E. Black Centre for Brain Resilience and Recovery, Toronto, ON, Canada; ^3^ Dr. Sandra Black Centre for Brain Resilience & Recovery, Sunnybrook Research Institute, Toronto, ON, Canada; ^4^ Dr. Sandra Black Centre for Brain Resilience and Recovery, Sunnybrook Research Institute, Toronto, ON, Canada; ^5^ Centre for Clinical Brain Sciences, The University of Edinburgh, Edinburgh, Scotland, United Kingdom; ^6^ University of Edinburgh and UK DRI, Edinburgh, United Kingdom; ^7^ UK Dementia Research Institute, University of Edinburgh, Edinburgh, United Kingdom; ^8^ Krembil Brain Institute, Toronto, ON, Canada; ^9^ Toronto Western Hospital, Toronto, ON, Canada; ^10^ Canadian Concussion Centre, Krembil Brain Institute, University Health Network, Toronto, ON, Canada; ^11^ Memory Clinic, Toronto Western Hospital, University Health Network, Toronto, ON, Canada; ^12^ Department of Psychiatry, University of Toronto, Toronto, ON, Canada; ^13^ Adult Neurodevelopment and Geriatric Psychiatry Division, CAMH, Toronto, ON, Canada; ^14^ Rotman Research Institute, Baycrest Academy for Research and Education, Toronto, ON, Canada; ^15^ Robarts Research Institute, University of Western Ontario, London, ON, Canada; ^16^ Physical Sciences, Sunnybrook Research Institute, Toronto, ON, Canada; ^17^ University of Toronto, Toronto, ON, Canada; ^18^ Division of Neurology, Department of Medicine, Sunnybrook Health Sciences Centre, Toronto, ON, Canada; ^19^ Department of Medical Imaging, University of Toronto, Toronto, ON, Canada; ^20^ Sunnybrook Research Institute, University of Toronto, Toronto, ON, Canada; ^21^ UK Dementia Research Institute, University of Edinburgh, Edinburgh, Scotland, United Kingdom; ^22^ University of Edinburgh, Edinburgh, United Kingdom; ^23^ Division of Neurology, Department of Medicine, University of Toronto, Toronto, ON, Canada; ^24^ Sunnybrook Research Institute, Toronto, ON, Canada; ^25^ Department of Medical Biophysics, University of Toronto, Toronto, ON, Canada; ^26^ Harquail Centre for Neuromodulation, Sunnybrook Research Institute, Toronto, ON, Canada

## Abstract

**Background:**

Enlarged perivascular spaces (PVS) are imaging biomarkers of cerebral small vessel disease (CSVD) associated with age, hypertension, and neurodegenerative conditions. Despite their clinical relevance, accurate quantification of PVS on T1‐weighted magnetic resonance imaging (MRI) remains challenging due to both variability across imaging protocols, and their small size and limited contrast. Automated methods such as convolutional neural networks (CNNs) offer a scalable solution, but existing tools are limited in performance and generalizability.

**Method:**

This study introduces segcsvd_PVS_, a CNN‐based tool designed for automated PVS segmentation on T1‐weighted images, based on a hierarchical framework incorporating anatomical information and robust training strategies. It was trained on semi‐automated RORPO‐based ground truth data and validated using both manual and semi‐automated segmentations. A large and comprehensive cohort (*n* = 1351) spanning multiple datasets characterized by diverse imaging protocols, patient populations, and anatomical characteristics was used for training and evaluation. Performance metrics, robustness to variations in image quality, and age‐related associations with PVS burden were rigorously evaluated against established RORPO‐based methods.

**Result:**

Segcsvd_PVS_ achieved high sensitivity for basal ganglia PVS (SNS = 0.81 ± 0.13) and identified significantly larger volumes (86.1 ± 67.1 mm^3^) compared to human tracers (47.2 ± 26.5 mm^3^, 48.6 ± 28.4 mm^3^. Our tool demonstrated strong age‐related correlations with PVS volumes across three diverse datasets (TEST: r = 0.41, CI = [0.03, 0.68]; ADNI: r = 0.38, CI = [0.30, 0.46]; CAHHM: r = 0.41, CI = [0.35, 0.46]). Although similar but weaker trends were observed for non‐basal ganglia PVS, segcsvd_PVS_ demonstrated superior robustness to variations in contrast and noise across both regions, with minimal changes in age‐related correlations (∆r ≤ 0.08) compared to the RORPO‐based methods (∆r ≤ 0.39).

**Conclusion:**

Segcsvd_PVS_ is a reliable tool for PVS segmentation, particularly in basal ganglia regions, offering superior sensitivity, robustness to imaging variability, and enhanced detection of biologically relevant age‐related associations. These findings support its application in large‐scale studies and clinical research to advance our understanding of PVS contributions to CSVD and dementia.